# On the neural origin of pseudoneglect: EEG-correlates of shifts in line bisection performance with manipulation of line length^☆^

**DOI:** 10.1016/j.neuroimage.2013.10.014

**Published:** 2014-02-01

**Authors:** Christopher S.Y. Benwell, Monika Harvey, Gregor Thut

**Affiliations:** a Centre for Cognitive Neuroimaging, Institute of Neuroscience and Psychology, University of Glasgow, Glasgow G12 8QB, UK; b School of Psychology, University of Glasgow, Glasgow G12 8QB, UK

**Keywords:** Pseudoneglect, EEG, Spatial bias, Attention, Landmark task, Event-related potentials

## Abstract

Healthy participants tend to show systematic biases in spatial attention, usually to the left. However, these biases can shift rightward as a result of a number of experimental manipulations. Using electroencephalography (EEG) and a computerized line bisection task, here we investigated for the first time the neural correlates of changes in spatial attention bias induced by line-length (the so-called line-length effect). In accordance with previous studies, an overall systematic left bias (pseudoneglect) was present during long line but not during short line bisection performance. This effect of line-length on behavioral bias was associated with stronger right parieto-occipital responses to long as compared to short lines in an early time window (100–200 ms) post-stimulus onset. This early differential activation to long as compared to short lines was task-independent (present even in a non-spatial control task not requiring line bisection), suggesting that it reflects a reflexive attentional response to long lines. This was corroborated by further analyses source-localizing the line-length effect to the right temporo-parietal junction (TPJ) and revealing a positive correlation between the strength of this effect and the magnitude by which long lines (relative to short lines) drive a behavioral left bias across individuals. Therefore, stimulus-driven left bisection bias was associated with increased right hemispheric engagement of areas of the ventral attention network. This further substantiates that this network plays a key role in the genesis of spatial bias, and suggests that post-stimulus TPJ-activity at early information processing stages (around the latency of the N1 component) contributes to the left bias.

## Highlights

• We examine the neural correlates of pseudoneglect in healthy participants using EEG.
• Pseudoneglect diminished with shortening of line length (line length effect).
• The ERP correlates localised to early right temporo-parietal junction activity.
• Right hemisphere engagement at early processing stages contributes to pseudoneglect.

## Introduction

Numerous lesion and neuroimaging studies have identified visuospatial attention processing to be predominantly lateralized to the right hemisphere (RH) of the human brain. RH dominance is evidenced by the more frequent and severe occurrence of visuospatial neglect after RH stroke as compared to left hemisphere (LH) stroke (Driver and Mattingley, 1998; Halligan et al., 2003; Harvey and Rossit, 2012; Karnath and Rorden, 2012; Mort et al., 2003; Parton et al., 2004; Vallar, 1998,) and by a substantial brain imaging literature in healthy participants (Cai et al., 2013; Cavezian et al., 2012; Çiçek et al., 2009; Fierro et al., 2001; Fink et al., 2000a,b, 2001; Foxe et al., 2003; Thiebaut de Schotten et al., 2005, 2011; Waberski et al., 2008). This hemispheric asymmetry is thought to underlie the tendency for the majority of neurologically normal individuals to display a behavioral bias in favor of stimuli appearing in their left visual field, a consistently reproduced phenomenon termed pseudoneglect (Bowers and Heilman, 1980; Charles et al., 2007; Jewell and McCourt, 2000; Sosa et al., 2010; Voyer et al., 2012).

The degree of pseudoneglect can be assessed using the horizontal line bisection task which is one of the most commonly employed metrics of lateralized spatial bias. During horizontal line bisection, healthy participants typically overestimate the length of the left end of the line (Jewell and McCourt, 2000). Interestingly, the degree of lateralized bias in visual processing is subject to dynamic changes within participants both in clinical and non-clinical populations, being modulated by experimental manipulation of non-spatial attentional factors such as time-on-task/arousal level (Bellgrove et al., 2004; Benwell et al., 2013; Dodds et al., 2008; Dufour et al., 2007; Fimm et al., 2006; Manly et al., 2005; Matthias et al., 2010; Newman et al., 2013; Robertson et al., 1998) and/or attentional/perceptual load (Bonato et al., 2010; Peers et al., 2006; Perez et al., 2008, 2009; Vuilleumier et al., 2008). With regard to non-clinical participants, prolonged time-on-task, reduced arousal and increased perceptual load all tend to result in a rightward shift in spatial bias and hence attenuation of the typical left bias (Bellgrove et al., 2004; Benwell et al., 2013; Dodds et al., 2008; Dufour et al., 2007; Fimm et al., 2006; Manly et al., 2005; Matthias et al., 2010; Newman et al., 2013; Perez et al., 2008, 2009). The rightward shift has been attributed to an interaction between spatial and non-spatial attention functions in the RH, localized respectively to the dorsal frontoparietal attention network (engaged in the control of spatial attention) and the ventral frontoparietal attention network (engaged in the maintenance of arousal and the detection of novel/salient stimuli). Depletion of processing capacity in the right lateralized ventral network under conditions of low arousal and/or high attentional load is postulated to reduce the left visual field advantage by causing right dorsal network deregulation (Corbetta and Shulman, 2002, 2011; Newman et al., 2013). This is in accordance with evidence that abnormal interactions between these networks also underlie the biased distribution of spatial attention in left neglect patients (Corbetta and Shulman, 2011; Corbetta et al., 2005, 2008; He et al., 2007).

Another factor which modulates the magnitude of bias (associated with line bisection decisions) is the length of the to-be-bisected line, again both in neglect patients (Anderson, 1996, 1997; Harvey et al., 1995; Mennemeier et al., 2005; Monaghan and Shillcock, 1998; Ricci and Chatterjee, 2001) and non-clinical samples (Benwell et al., 2013; Heber et al., 2010; McCourt and Jewell, 1999; Mennemeier et al., 2005; Rueckert et al., 2002; Thomas et al., 2012). Recent studies in healthy participants, employing a perceptual computerized line bisection task (the landmark task (Harvey et al., 2000; Milner et al., 1992; Olk and Harvey, 2002)), have shown that while long lines (subtending > 6° horizontal visual angle (VA) in length) induce a systematic (usually left) bias, short lines (subtending < 2° VA) induce no such consistent bias (Benwell et al., 2013; Heber et al., 2010; Thomas et al., 2012) or can even be associated with a significant right bias when combined with manipulation of non-spatial attention through extended time-on-task (Benwell et al., 2013). Hence, as with manipulation of non-spatial attention, manipulation of line length leads to a rightward shift in spatial bias. The line length effect has been hypothesized to arise from asymmetrical hemispheric contributions (RH > LH) to the perceived salience of visual stimuli that is more pronounced for peripheral stimuli or stimulus-parts stretching into the peripheral visual field, hence a left bias arises more prominently for long rather than short lines (Anderson, 1996; Benwell et al., 2013; McCourt and Jewell, 1999; Monaghan and Shillcock, 1998, 2004).

While a right hemispheric dominance in spatial attention and a left spatial bias in visual processing are well documented, and the attenuation of left visual field bias with depletion of right hemispheric function would suggest a close link, relatively little is known about the information processing stages during which the bias arises. Only a handful of electroencephalographic (EEG) studies have looked at the neural correlates of line bisection per se in healthy participants (Foxe et al., 2003) and at the neural correlates of the above rightward shifts in bias in particular (for manipulation of time-on-task see Newman et al., 2013; for perceptual load see O'Connell et al., 2011; Perez et al., 2009). Also, while spatial bias is shifting rightward with both manipulation of non-spatial attention (such as perceptual load and arousal) and stimulus properties (line length), it is unclear whether one common mechanism underlies both of these changes, or alternatively whether they are determined at distinct processing stages. Unlike for perceptual load (O'Connell et al., 2011; Perez et al., 2009) and time-on-task (Newman et al., 2013), to date the neural correlates of the effect of line length have not been investigated empirically using EEG. Elucidating the neural correlates of different experimental manipulations of lateralised spatial bias within participants and whether these are driven by the same or different mechanisms should help in understanding more fully the functional architecture of the visuospatial attention system.

In the present EEG study, we aimed to determine, for the first time, the neural correlates of the line length effect in healthy participants (rightward shift in line bisection performance with decreasing line length), and to interpret this in light of previous EEG studies on the neural correlates of line bisection performance per se (e.g. Foxe et al., 2003) and the rightward shift in spatial bias with manipulation of non-spatial attention (O'Connell et al., 2011). Foxe et al. (2003) investigated the event related potential (ERP) correlates of line bisection decisions and reported right lateralized activity with source estimates in temporo-parietal junction (TPJ) during the early phase of bisection decisions (at the latency of the N1 component), followed by right superior parietal activity (in the vicinity of the intraparietal sulcus (IPS)), in good agreement with fMRI-signatures of landmark task processing (Cai et al., 2013; Cavezian et al., 2012; Çiçek et al., 2009; Fink et al., 2000a, 2000b, 2001). O'Connell et al. (2011) examined the neural correlates of the rightward shift in bias with increased attentional load using ERPs. Employing a lateralized target detection paradigm with a simultaneous central alphanumeric target detection task, the authors manipulated the level of attentional load required at fixation. Compared to the low central load condition (unique feature detection), high central attentional load (detection of conjunction of features) led to an attenuation of the RH–N1 response to contralateral stimuli (O'Connell et al., 2011). Interestingly, the effect again source-localized to regions of the right TPJ: a key node in the ventral frontoparietal attention network thought to determine spatial bias in interaction with the dorsal attention network (Corbetta and Shulman, 2002, 2011). The implication of the N1 component in the genesis of the spatial bias is in line with previous single pulse transcranial magnetic stimulation (TMS) studies in healthy participants (Dambeck et al., 2006; Fierro et al., 2001) and ERP studies of altered visuospatial processing in neglect patients (Di Russo et al., 2008; Tarkka et al., 2011). Based on the above studies, we hypothesized that attenuation of right lateralized TPJ-activity at the latency of the N1 component may also be a good candidate signature of the rightward shifts seen in line bisection with manipulation of line length. In line with this, we found the effect of line length to be reflected predominantly during the N1 over the RH in regions of the TPJ (source estimates). Furthermore, our data reveal how neural activity maps onto shifts in behavioral bias. The attenuation of the N1 component over the RH by line length (long v short) was found to correlate with the associated rightward shift in behavioral bias across participants. Hence, the neural correlates of rightward shifts in attention through manipulation of central attentional load (see O'Connell et al., 2011) versus manipulation of line length (the present study) have much in common, both in terms of their anatomy (right TPJ) and timing (N1). Implications for understanding the processes involved in the rightward attentional shift with these manipulations are discussed.

## Method

Participants were asked to perform two tasks on lines of two different lengths: A) the landmark task and B) a control condition in which they simply indicated if the presented line was transected or not (task manipulation adopted from Foxe et al., 2003; Waberski et al., 2008). This served to examine the electrophysiological correlates of line bisection performance and differences in the bisection bias induced by the line length modulation. Note that the focus on the line length effect emphasizes the contrast between bisection of long lines and bisection of short lines (long minus short lines) within the same participant. As well as providing the means for investigating the neural correlates of the line length effect itself, this manipulation effectively corrects for individual factors influencing the EEG signal measured over the scalp (such as overall activation influenced by arousal level and individual differences in skull thickness and volume conduction). This correction is important as these factors may confound the scalp signals and render the comparison of ERP amplitudes between participants, and in particular brain–behavior correlations across participants, problematic.

### Participants

Nineteen right-handed participants (14 male, 5 female, mean age = 24.14 years, max = 30 years, min = 20 years) received financial compensation for their participation in the experiment. However, due to poor behavioral performance on the landmark task (see the Behavioral analysis of landmark task performance section), 2 participants were excluded from the final analysis. Written informed consent was obtained from each participant. All participants were volunteers naive to the experimental hypothesis being tested. All participants reported normal or corrected to-normal vision and no history of neurological disorder. The experiment was carried out within the Institute of Neuroscience and Psychology at the University of Glasgow and was approved by the local ethics committee.

### Instrumentation and stimuli

**Fig. 1:**
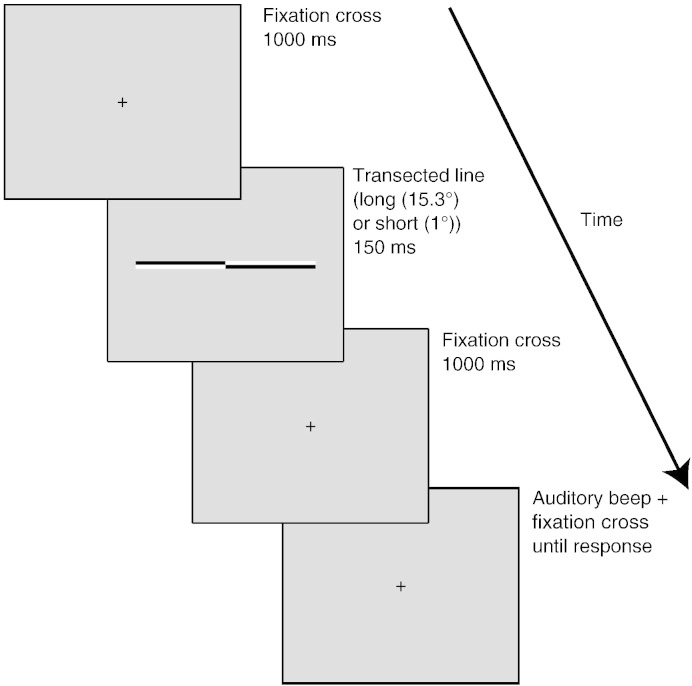
Experimental paradigm and sequence of events during each trial. Each trial was initiated by the appearance of a fixation cross for 1000 ms followed by presentation of the line stimulus for 150 ms followed by the fixation cross, which remained on the screen until the end of the trial. Participants were requested to delay their manual response for 1000 ms following the presentation of the stimulus in order to obviate for motor artifacts in the EEG signal. The onset of the response period was indicated by an auditory beep (100 Hz). In landmark task blocks, participants were asked to judge which end of the pre-transected line appeared shortest. The long line displayed is veridically transected but lines could be transected at any 1 of 29 points ranging symmetrically from ± 4.36% of absolute line length to veridical center. Long (15.3° × .39°) and short (1° × .39°) lines were presented in separate blocks. In control task blocks, 25% of the presented lines were not transected (plain white lines) and participants were asked to indicate whether the line was transected or not.

Stimuli were presented using the E-Prime software package (Schneider et al., 2002) on a CRT monitor with a 1280 × 1024 pixel resolution and 85 Hz refresh rate. Adapted from McCourt (2001) and Benwell et al. (2013), the paradigm represented a computerized version of the landmark task. Lines of 100% Michelson contrast were presented on a gray background (luminance = 179, hue = 179). Fig. 1 shows an example of the line stimuli used in the experiment as well as a schematic of the trial procedure. Two different line lengths were presented. At a viewing distance of 100 cm, ‘long’ lines subtended 15.3° (width) by .39° (height) VA. At the same viewing distance, ‘short’ lines subtended 1° by .39° VA. For the line bisection task, lines were transected at 1 of 29 points ranging symmetrically from ± 4.36% of absolute line length to veridical center. All lines were displayed with the transector location centered on the vertical midline of the display (i.e., aligned to a central fixation cross which preceded the presentation of the lines, see below). For the non-spatial control task (judge whether line is transected or not), non-transected lines were intermixed with the same bisected lines used for the line bisection task.

### Procedure and task

Subjects were seated in a comfortable chair 100 cm from the display monitor, their midsagittal plane aligned with the center of the screen. Subjects performed two different tasks during the experiment: A landmark task in which they were asked to judge which of two ends of a pre-transected line was shorter (left or right response) and a control task in which they were asked to judge simply whether a line was transected or not. Each subject performed 4 blocks overall (174–180 trials per block): 1 block of the landmark task with long lines (Long Line LM (LL LM)), 1 block of the landmark task with short lines (Short Line LM (SL LM)), 1 block of the control task (Long Line Control (LL C)) and 1 block of the control task with short lines (Short Line Control (SL C)). Each block took 8–10 min to complete. The order of block performance was counter-balanced across subjects.

#### Landmark task

During landmark task performance (both for long and short lines) (see also Fig. 1), each trial began with presentation of a fixation cross (.39° height × .39° width) for 1 s followed by presentation of the transected line (150 ms). The transection mark was always aligned with the fixation cross (i.e., the eccentricity of the line endpoints varied across trials while the transection point always appeared at the same central position), therefore preventing use of the position of the fixation cross relative to the bisection mark, as a reference point for bisection judgments. Following the disappearance of the line, the fixation cross returned. Participants were instructed to delay their response for 1 s until they heard an auditory beep in order to obviate motor artifacts in the EEG signal. During the response period following the beep, participants indicated their judgment of which end of the line was shorter (which end of the line the transection mark appeared closest to) by pressing either the left or right response key on a keyboard. Participants always responded using their dominant right hand (right index and middle fingers respectively). Participants were instructed to hold their gaze on the center of the screen throughout each trial and to try to keep eye blinks/movements to a minimum. The subsequent trial began as soon as the response was made. Trials lasted between 2 and 3 s. Trial type (transector location) was selected at random within a block. During landmark task blocks, participants made 6 “left–right” judgments at each transector location (29 locations) such that estimates of perceived line midpoint were based on 174 trials.

#### Control task

During control task performance (both for long and short lines), trial structure was identical to the landmark task (see Fig. 1). Each trial began with presentation of a fixation cross (.39° height × .39° width) for 1 s followed either by presentation of a transected line (75% of trials) or a line of the same length with no transection mark (25% of trials) for 150 ms. Following the disappearance of the line the fixation cross returned. Again, participants were instructed to delay their response for 1 s until they heard an auditory beep. During the response period following the beep, subjects indicated their judgment of whether the line had contained a transection mark or not, by pressing either the left (transection mark present) or right (no transection mark) response key on the keyboard. The subsequent trial began as soon as the response was made. Trials lasted 2–3 s. Trial type (transector location and line type (plain v transected)) was selected at random within a block. This control task has been employed previously to dissociate EEG activity related to line bisection performance from that of an attentionally demanding non-spatial judgment (Foxe et al., 2003; Waberski et al., 2008) and allows for equivalent button presses during landmark and control task performance. Control blocks consisted of 176 trials.

### Behavioral analysis of landmark task performance

In order to obtain an objective measure of perceived line midpoint for both long and short lines in each participant, psychometric functions (PFs) were derived using the method of constant stimuli. The dependent measure was the proportion of trials on which participants indicated that the transector had appeared closer to the left end of the line. Non-linear least-squares regression was used to fit a cumulative logistic function to the data for each line length in each participant. The cumulative logistic function is described by the equation:

$$ƒ\left(\mu ,x,s\right)=1/\left(1+\exp\left(\left(x-\mu \right)/s\right)\right)$$

where x are the tested transector locations, μ corresponds to the x-axis location with 50% ‘left’ and 50% ‘right’ response rates and s is the estimated width of the psychometric function. The 50% location is known as the point of subjective equality (PSE) and represents an objective measure of perceived line midpoint. The width of the PF provides a measure of the precision of participants' line midpoint judgments per block. On the basis of extreme curve width values, 2 participants were excluded from further behavioral and EEG analyses: the curve widths of each of these subjects for both long and short lines were flagged as outliers by application of the median absolute deviation (MAD) rule for outlier detection. Inferential statistical analyses were performed on the individually fitted PF PSE values of the remaining 17 subjects. To test for a systematic directional bias in each condition, long and short line PSE values were separately compared to 0 (representing the veridical center of the line) using a nonparametric 1-sample Wilcoxon signed rank test.

### Electrophysiological measures

Continuous electroencephalogram (EEG) recording was acquired from each participant at 1000 Hz through 62 scalp electrodes and 4 ocular electrodes (horizontal and vertical bipolar montage), with impedances < 10 kΩ (Brain Products). ERP-analysis was conducted using the EEGLAB toolbox (Delorme and Makeig, 2004) and the Mass Univariate ERP Toolbox (Groppe et al., 2011a). Source estimates were calculated using Cartool (Brunet et al., 2011; http://sites.google.com/site/fbmlab/cartool). Offline, the channel mean was removed from each channel, data de-trending was performed, a 0.3 high-pass filter and a 40 Hz low-pass filter were applied and the data were epoched between − 500 and 1000 ms pre- to post-stimulus onset. Thereafter, trials with abnormal activity (extreme value rejection criterion of ± 60 μV) or horizontal eye movements (based on horizontal electrooculogram) were rejected and bad channels were removed without interpolation. An independent component analysis (ICA) (Delorme and Makeig, 2004) was run using the runica EEGLAB function (Delorme and Makeig, 2004; Delorme et al., 2007) and components corresponding to blink activity were removed. Subsequently, data were re-epoched between − 300 and 500 ms and baseline corrected (− 300 ms to 0). Finally, previously rejected channels were interpolated using a spherical spline interpolation and the data were recalculated against the average reference. Responses to the non-transected lines in the control condition were not included in the EEG analysis as they did not appear during landmark task blocks. Consequently, the number of trials entered into the grand average per participant was equated to a common minimal denominator across conditions (taking into account only transected and artifact-free trials) which amounted to the following average number of trials entered into the grand average per condition: LL LM (103.7059 (min = 83, max = 120)), SL LM (104.1765 (min = 78, max = 123)), LL C (105.9412 (min = 89, max = 116)), and SL C (104.9412 (min = 96, max = 115)).

### Mass univariate EEG analysis

We sought to dissociate the effect of line length (long v short lines) from the effect of task (landmark task v control task) on event-related potentials (ERPs), and to investigate whether an interaction exists between the two. To this end, main effects of line length were quantified by comparing long- versus short-line ERPs collapsed across landmark and control tasks. Then, the main effects of task were quantified by comparing landmark-task and control-task ERPs collapsed across long and short lines. Finally, line-length × task interaction effects were quantified by calculating the difference between long and short lines and comparing this difference between tasks (landmark task versus control task; double difference). Periods of amplitude modulation between conditions were identified using pairwise comparisons at each time point across all electrodes. This analysis was carried out separately for each of the two main effects (hereafter referred to as the “line length effect” and “line bisection effect”), as well as for the interaction between the two. In order to control the familywise error rate (FWER), cluster-based permutation tests (Bullmore et al., 1999; Groppe et al., 2011b; Maris and Oostenveld, 2007) were employed. The calculation of the test statistic involved the following: Based on the initial pairwise comparisons, all t-scores falling below a threshold corresponding to an uncorrected p-value of 0.01 were ignored. The remaining t-scores were formed into clusters by grouping together t-scores at adjacent time points and electrodes (this step was performed separately for samples with positive and negative t-values (two-tailed test)). The spatial neighborhood of each electrode was defined as all electrodes within approximately 3.65 cm, resulting in a mean of 3.5 (max = 4, min = 1) and median of 4 neighbors per electrode. The t-scores of each cluster were subsequently summed to produce a cluster–level t-score. The most extreme cluster–level t-score across 20,000 permutations of the data was used to provide a data driven null hypothesis distribution. The relative location of each observed cluster level t-score within the null hypothesis distribution indicates how probable such an observation would be if the null hypothesis were true (no ERP difference between conditions). A 1% alpha level was adopted in order to strengthen familywise error rate control.

### Hemispheric asymmetry EEG analysis

In order to probe hemispheric lateralization in the ERPs related to the line length and the line bisection effects, the electrode showing the largest sensitivity to each manipulation (as indexed by the largest t-score) and the equivalent contralateral electrode were selected from the time periods specific to each effect. The mean amplitudes at these electrodes during the time periods of differences between conditions were averaged and entered into a 2 × 2 (hemisphere × line length / task) repeated measures analysis of variance (ANOVA).

### Correlation analysis

To further investigate the relationship between the bisection bias and right hemispheric activity related to the effects of line length or task (which both proved to be right lateralized, see the Results section), separate correlation analyses were carried out between peak ERP amplitude and bisection bias during time periods associated with both the line length effect and the line bisection effect. As behavioral measures, we entered the individual differences in behavioral bias associated with the difference in line length (i.e. PSE for long lines minus PSE for short lines) into the correlation analyses. This subtraction between conditions (but within participants) controls for inter-individual differences in arousal levels potentially confounding the behavioral bias, because bias per se depends on time-on-task whereas the relative bias (long minus short) does not (see Benwell et al., 2013). Behavioral biases were measured in pixels relative to veridical line center. These values were correlated with the corresponding individual ERP difference associated with the line length effect or alternatively, with the individual ERP difference associated with the line bisection effect. To determine individual ERP differences associated with the line length manipulation, the electrode showing the strongest line length effect (as indexed by the highest t-score) was selected and its peak amplitude during the line length effect period extracted for both conditions from each participant and then subtracted (long lines minus short lines). Likewise, to obtain an individual measure of ERP activity specific to line bisection, the electrode showing the strongest line bisection effect was selected, peak amplitudes (during the line bisection effect period) extracted for both conditions per participant, and then subtracted (landmark task minus control task). In analogy to behavior, subtracting ERP data between conditions (but within participants) effectively corrects for inter-individual differences potentially confounding the EEG signals, such as arousal, skull thickness or volume conduction. Both Pearson's r and Spearman's rho were calculated for each correlation (with their 95% percentile bootstrap confidence intervals) in order to attain robust measures of association.

### Source estimates and analysis

We estimated the localization of the electrical activity in the brain using a distributed linear inverse solution (minimum norm) applying the LAURA regularization approach comprising biophysical laws as constraints (Grave de Peralta et al., 2004, 2001 and Michel et al., 2004). LAURA selects the source configuration that better mimics the biophysical behavior of electric vector fields (i.e. activity at one point depends on the activity at neighboring points according to electromagnetic laws). LAURA was implemented in a realistic head model using 4024 nodes, selected from a 6 × 6 × 6 mm grid equally distributed within the gray matter of the Montreal Neurological Institute's average brain. To estimate the source of the line length effect and the line bisection effect, we performed statistics on the source estimations in the time periods associated with the effect of line length (100–200 ms; comparing source estimates of long lines versus short lines, collapsed over tasks) and with the effect of task (230–500 ms; comparing source estimates of line bisection versus control task, collapsed over stimuli).

## Results

### Behavioral results

**Fig. 2:**
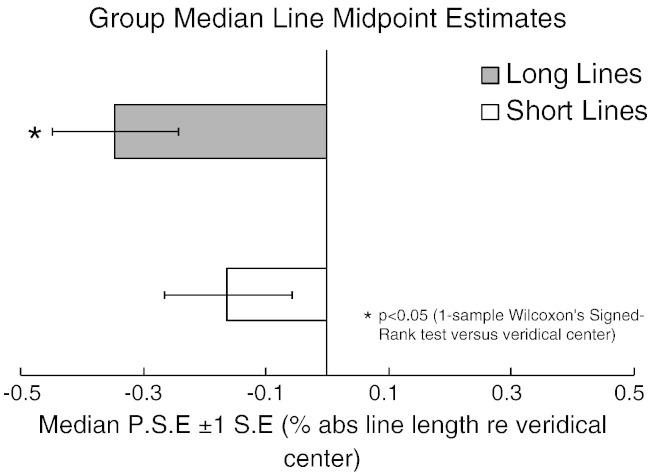
Behavioral bias data. Group-averaged (N = 17) point of subjective equality (± 1 SE) for both long (gray bar) and short (white bar) landmark task performance (in % of absolute line length relative to veridical center). Negative values indicate leftward bias. Note the typical systematic leftward error (pseudoneglect) is stronger for long than short lines.

Fig. 2 presents the median of individually fitted PSE's (% of absolute line length relative to veridical center ± 1 SE) as a function of line length. In long lines, median PSE was displaced to the left of veridical center by − 0.35%, and this bias was significantly different from veridical center (0) (Wilcoxon's signed-rank test, p = .044) indicating a systematic leftward bias. In short lines, median PSE was displaced to the left of veridical center by − 0.15%, but this was not significantly different from veridical center (0) (Wilcoxon's signed-rank test, p = .554) indicating no systematic bias in short lines.

### EEG results

**Fig. 3:**
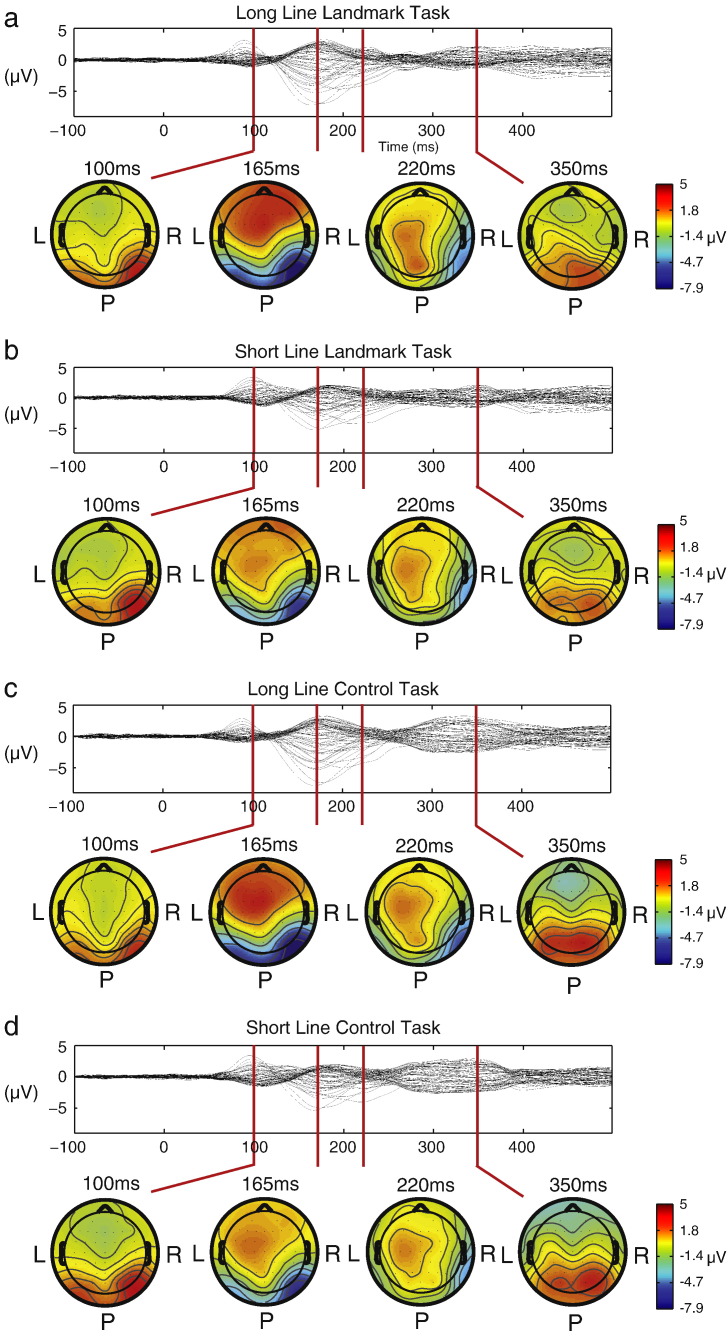
Group-averaged (N = 17) voltage waveforms (62-channel butterfly plot) and topographic maps at selected time points corresponding roughly to the traditional P1, N1, P2 and P3 components of the ERP. Data are shown separately for (a) long line landmark task, (b) short line landmark task, (c) long line control task and (d) short line control task performance. L: Left, R: right, P: posterior.

**Fig. 4:**
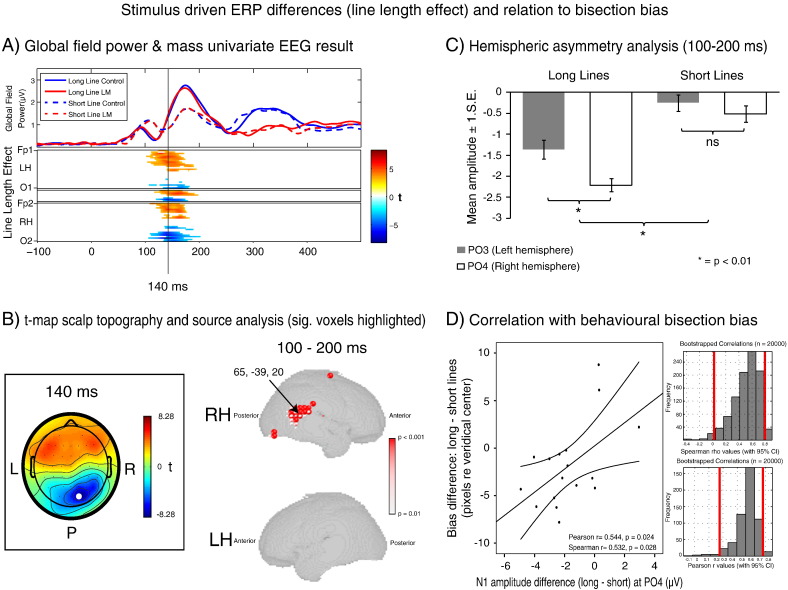
Line-length EEG-effects. (A) Global field power (GFP) over time for each experimental condition (upper panel) and mass univariate analysis results of the line-length effect (lower panel). Note in the GFP the early grouping of conditions according to line length (red & blue solid lines vs. red & blue dashed lines: i.e. long vs. short lines). The corresponding mass univariate analysis revealed these differences to be significant, peaking at 140 ms post-stimulus onset. (B) Topographical t-map (long minus short) across the scalp at 140 ms (left panel) and source estimate p-value maps of the effect (right panel, only p-values reaching a significance level of p < 0.01 are displayed, p-value coded by voxel size and color). Note that the line length effect peaked at electrode PO4 (electrode marked in white) and localized to the temporo-parietal junction of the RH (max. significant voxel: Talairach coordinate: 65, − 39, 20, peak t-value = 4.59, p < 0.001). (C) Hemispheric asymmetry data for electrodes PO3/PO4. Long lines were associated with a hemispheric asymmetry (RH > LH), not present in short lines. (D) Relationship between the line-length effect in ERPs (long–short lines) at PO4 and the line-length effect in behavioral bias (long–short lines) across individuals (left panel), and histograms of the corresponding Pearson and Spearman bootstrapped correlation values (right panels, red bars = 95% confidence intervals). The correlation proved significant by both correlation methods (p < 0.05) and the bootstrapped 95% confidence intervals for both did not include 0. The positive relationship suggests that the level to which the RH is engaged by “long” line processing during the early time period influences the direction and magnitude of lateralized behavioral bias.

**Fig. 5:**
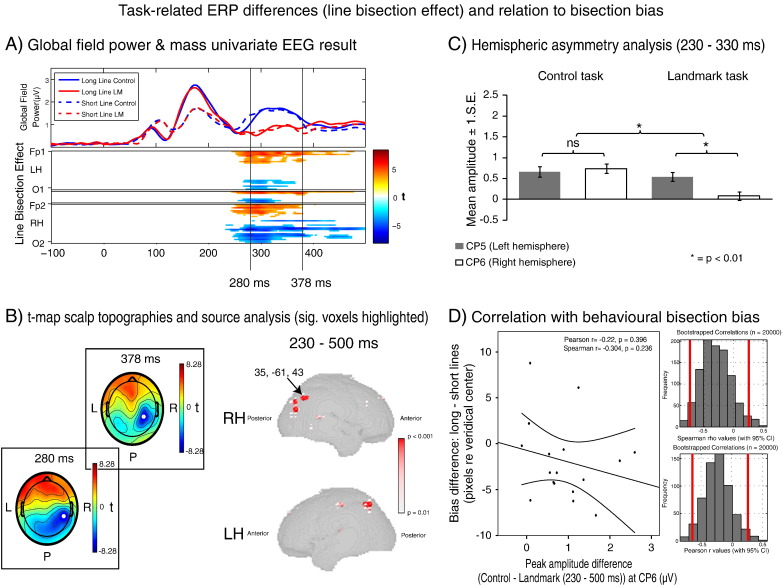
Line-bisection EEG-effect. (A) Global field power (GFP) over time for each experimental condition (upper panel) and mass univariate analysis results of the line bisection effect (lower panel). Note in the GFP the late grouping of conditions according to task (red lines vs. blue lines: i.e. bisection vs. control task). The corresponding mass univariate analysis revealed these differences to be significant, peaking at 280 ms and 378 ms post-stimulus onset. (B) Topographical t-maps (control minus landmark task) across the scalp at 280 ms and 378 ms (left panel) and source estimate p-value maps of the effect (right panel). Note that the line bisection effect peaked at electrodes CP6 (280 ms) and CP4 (378 ms), shown in white, and localized largely to the right superior parietal cortex (max. significant voxel: Talairach coordinate: 35, − 61, 43 (peak t-value = − 3.3, p < 0.01)). (C) Hemispheric asymmetry data for electrodes CP5/CP6. Landmark task performance was associated with a hemispheric asymmetry, not present during the control task. (D) Relationship between the line bisection effect in ERPs (control–landmark tasks) at CP6 and the length effect in behavioral bias (long–short lines) across individuals (left panel) and histograms of the corresponding Pearson and Spearman bootstrapped correlation (right panel, red bars = 95% confidence intervals). The correlation was not significant for either correlation method (p > 0.05) and the bootstrapped 95% confidence intervals for both included 0.

The group averaged visual evoked potentials for all electrodes (from − 100 to 500 ms relative to stimulus onset) are presented as butterfly plots separately for all four conditions in Fig. 3. Also presented are group averaged topographies at time points corresponding to the traditional P1, N1, P2 and P3 series of ERP components. Figs. 4A and 5A illustrate the corresponding global field power (GFP) plots for each of the four conditions (upper left panels, information duplicated in Figs. 4 and 5 for a better comparison with the respective mass univariate results illustrated below). These plots clearly reveal an early grouping of condition according to line length (red & blue solid lines vs. red & blue dashed lines: i.e. long vs. short lines) which occurs between 100 and 200 ms post-stimulus onset. In a later time window (300–400 ms), these conditions regroup according to task (red lines vs. blue lines: i.e. bisection vs. control task). The corresponding mass univariate analysis revealed these differences to be significant. The line length effect (long versus short lines, Fig. 4A) preceded the line bisection effect (line bisection versus control line judgment task, Fig. 5A) with no overlap of the two effects in time. In addition, no significant interaction effect between line-length and task was found at any time point during the epoch (not shown). Below, we first present the results of the line length effect and of the follow-up analyses on source estimates and the relation to behavior (Line length effect section), before presenting the line bisection effect and follow-up analyses (Line bisection effect section).

#### Line length effect

Fig. 4A (lower panel) presents the results of the mass univariate analysis of the effect of line length across time (x-axis) over electrodes (y-axis: anterior–posterior electrodes, broken down by left vs. right hemisphere: LH vs RH). The analysis revealed significant differences between long and short lines in terms of ERP amplitude from 102 to 202 ms post-stimulus onset over posterior electrodes (increased negativity in long lines compared to short lines, coded in blue tones) and frontal electrodes (increased positivity in long lines compared to short lines, coded in red tones). The peak of the effect (in terms of t-score) occurred 140 ms post-stimulus onset at RH parieto-occipital electrode PO4 (t-score = − 8.28, time point marked in Fig. 4A). Fig. 4B (left map) illustrates the topographical distribution of t-scores (long minus short) across the scalp at the selected time point (electrode of maximum difference between conditions shown in white). As well as being strongest over the RH, the increased negativity in long lines over posterior (occipital, parietal and central–parietal) electrodes was also more widespread over the right hemisphere as compared to the left hemisphere (see Fig. 4B). It is important to note that this right lateralized topography with a posterior maximum was stronger for long than short lines (line length effect) irrespective of task, as no interaction with task was found (see above), i.e. this occurred independently of whether the line needed to be mentally bisected or not.

##### Line length topography: Hemispheric lateralization

To probe for hemispheric lateralization of the line length topography (not tested by the electrode-wise mass univariate analysis above), we subjected the mean ERP amplitude at homologous electrodes of maximum line length effects (PO3 vs PO4, 100–200 ms) to a 2 (line length: long vs short) × 2 (hemisphere: PO3 left vs PO4 right) repeated measures ANOVA. The corresponding data are shown in Fig. 4C. The ANOVA revealed a significant main effect of line length [F(1, 16) = 29.534, p < .001], no significant main effect of hemisphere [F(1, 16) = 4.056, p = .061] and a significant line length × hemisphere interaction [F(1, 16) = 10.176, p = .006]. Analysis of simple main effects (paired-sample t-tests performed between hemispheres for long and short lines separately) revealed that long lines induced a hemispheric asymmetry, with an increased negativity in the RH as compared to the LH [t(16) = 2.561, p = .021]. No significant difference in amplitude was observed between hemispheres during short line processing [t(16) = 1.080, p = .296]. This supports right hemispheric lateralization of the line length topography.

##### Line length effect: Correlation with behavioral bias across participants

The correlation analysis between the line length effect in RH-ERP (PO4) and the spatial bisection bias across individuals revealed a positive correlation in both Pearson [Pearson r = 0.544, p = 0.024] and Spearman analysis [Spearman r = 0.532; p = 0.028] and proved robust for both when bootstrapped [Pearson correlation: bootstrap 95% CI = 0.142, 0.741, Spearman correlation: bootstrap 95% CI = 0.022, 0.799] (see Fig. 4D). The larger the difference in RH peak (N1) amplitude over electrode PO4, the larger the difference in landmark task bias between short and long lines. This suggests that the level to which the RH is engaged during this time period (100–200 ms) influences the direction and magnitude of the lateralized behavioral bias.

##### Line length effect: Source estimates

In source space, voxels with maximum significant differences between the two line length conditions (long vs short lines) in the relevant time interval (100–200 ms) were localized to the RH (see Fig. 4B). The source estimates implicated the right inferior parietal cortex and the right superior temporal sulcus in the line length effect indicating that regions of the right temporo-parietal junction (TPJ) were the likeliest generators of the line-length effect in sensor space (max. significant voxel: Talairach coordinate: 65, − 39, 20 (peak t-value = 4.59, p < 0.001)).

#### Line bisection effect

Fig. 5A (lower panel) illustrates the results of the mass univariate analysis of the effect of task (line bisection vs. control) in a time (x-axis) × electrode (y-axis) plot. The analysis revealed significant differences between line bisection and control task in terms of ERP amplitude from 231 to 500 ms post-stimulus onset over (mainly RH lateralized) centro-parietal electrodes (increased negativity in the landmark task compared to the control task, coded in blue tone) and a more widely spread difference over frontal electrodes (increased positivity in the landmark task compared to the control task, coded in red tones). Notable peaks (in terms of t-scores) were present at 280 ms post-stimulus onset at RH centro-parietal electrode CP6 (t-score = − 7.36), and at 378 ms post-stimulus onset at RH centro-parietal electrode CP4 (t-score = − 7.173, time points marked in Fig. 5A). Fig. 5B (left map) shows the topographical distribution of t scores (line bisection minus control) across the scalp at these time points (electrodes of maximum difference between conditions shown in white). Both of these topographies revealed increased RH negativity in line bisection as compared to the control task, and their maxima were located in a more superior position than the RH negativity of the line length topography (compare Figs. 5B vs. 4B, centro-parietal vs occipito-parietal positions).

##### Line bisection topography: Hemispheric lateralization

To probe for hemispheric lateralization of the line bisection topography (in analogy to the above analysis on line length effects), we subjected the corresponding data to 2 (task: line bisection vs control) × 2 (hemisphere: left vs. right CP electrodes) repeated measures ANOVAs. The 2 × 2 ANOVA on CP5/CP6 (230 ms–330 ms, data shown in Fig. 5C) revealed a significant main effect of task [F(1, 16) = 11.721, p = .003], no significant main effect of hemisphere [F(1, 16) = .998, p = .333] and a significant task × hemisphere interaction [F(1, 16) = 7.893, p = .013]. Analysis of simple main effects (paired-sample t-tests performed between control and line bisection task for the LH and the RH separately) revealed an increased negativity in the bisection task as compared to the control task in the RH/CP6 [t(16) = 3.664, p = .002] but no significant difference between the two in the LH/CP5 [t(16) = 1.556, p = .139]. Likewise, the 2 × 2 ANOVA on CP3/CP4 (330–500 ms, data not shown) revealed a significant main effect of task [F(1, 16) = 26.005, p < .001], no significant main effect of hemisphere [F(1, 16) = 1.031, p = .325] and a significant task × hemisphere interaction [F(1, 16) = 7.627, p = .014]. Analysis of simple main effects again revealed an increased negativity in the bisection task compared to the control task in the RH/CP4 [t(16) = 6.179, p < .001] but no significant difference in the LH/CP3 [t(16) = .831, p = .418].

##### Line bisection effect: Correlation with behavioral bias across participants

The above analysis of line bisection effects shows a stronger right lateralized centroparietal negativity during line bisection as compared to the non-spatial control task in the later window of the epoch. However, this right lateralization occurred for line bisection independently of line length (no interaction with line length, see above), suggesting that activity during this time period is unlikely to account for pseudoneglect. In line with this view, ERP activity in this time window was not correlated with behavioral bias (see Fig. 5D). The correlation analysis between the line bisection effect in RH-ERP (CP6) and the spatial bisection bias across individuals revealed no significant association [Pearson r = − 0.220, bootstrap 95% CI = − 0.667, 0.270, p = 0.396; Spearman r = − 0.304, bootstrap 95% CI = − 0.744, 0.252, p = 0.236].

##### Line bisection effect: Source estimate

Voxels with maximum significant differences between the two tasks (bisection vs control task) in the time interval associated with the line bisection effect (230–500 ms) were again localized to the right hemisphere, but with a more superior localization (see Fig. 5B). The source estimates implicated the right superior parietal cortex in the bisection effect. The maximum significant difference was observed at 35, − 61, 43 (Talairach coordinate (peak t-value = − 3.3, p < 0.01)), in the vicinity of the intraparietal sulcus (IPS).

## Discussion

We studied the neural underpinning of the line length effect in line bisection for the first time using stimulus-locked ERPs. Behaviorally, we found that most participants displayed a systematic leftward bias (pseudoneglect) during long line landmark task performance whereas no systematic bias was observed during performance of the task with short lines, in line with the previously reported line-length effect (Benwell et al., 2013; Heber et al, 2010; McCourt and Jewell, 1999; Rueckert et al., 2002; Thomas et al., 2012). Our EEG findings establish that an increased engagement of areas of the right lateralized, ventral attention network contributes to the genesis of the spatial bias, and that this engagement is stimulus-driven (task independent because it was observed in both the line bisection and control tasks): We found an ERP response which showed higher amplitude to long than short lines, corresponded in timing to the N1-component and was right lateralized to areas of the temporo-parietal junction. Furthermore, the difference in peak N1-amplitude between long and short line processing correlated with the difference in line bisection bias between long and short lines across participants.

### Neural (ERP) substrates for behavioral line bisection bias

Our findings, in combination with those of O'Connell et al. (2011), suggest a common neural substrate for the rightward shifts in behavioral bias observed with decreased line length and increased perceptual load respectively: Both experimental manipulations were associated with an attenuation of right-lateralized TPJ activity at the latency of the N1-component (O'Connell et al., 2011 and the present study). Extending the results of O'Connell et al. (2011), we here show in addition that the degree of this attenuation correlates with the degree of the rightward shift in behavioral bias across participants. Overall, this provides further evidence that pseudoneglect can be attributed to the predominant role played by the RH in visuospatial processing (as initially suggested by Heilman and Van Den Abell, 1980; Mesulam, 1981 and later Reuter-Lorenz et al., 1990; Bultitude and Aimola-Davies, 2006) and to areas of the right ventral attention network in particular (Corbetta and Shulman, 2002, 2011; Newman et al., 2013) especially when processing involves stimuli appearing/stretching into the periphery of the visual fields. This is in line with mathematical models of the relative hemispheric contributions to the perceived salience of visual stimuli (RH > LH) (Anderson, 1996; Monaghan and Shillcock, 1998, 2004 and see the discussion in Benwell et al., 2013). In light of these models, our results would suggest that the asymmetric hemispheric contribution (in favor of the RH) to the salience–perception of lateral visual stimuli can be attributed to an increased activation of areas around the right-TPJ (compared to the left) in long lines that is not present in short lines.

The question then arises as to why line length modulates the degree to which the right TPJ is activated, and how this would fit with the notion of the interplay between the right ventral arousal/re-orienting network and dorsal spatial attention network modulating spatial bias (Corbetta and Shulman, 2011; Corbetta et al., 2005, 2008; He et al., 2007; Thiebaut de Schotten et al., 2011). Given that the right TPJ is preferentially activated during long line processing, we conclude that the resources of the RH ventral network may be less engaged in processing short lines. It is conceivable that short lines may be less attentionally ‘salient’ and so activate the ventral network less strongly. Although this would not constitute a depletion of processing capacity in the RH ventral network with reduced line length (such as presumably achieved by increased foveal perceptual load and reduced arousal/time-on-task; see Bellgrove et al., 2004; Benwell et al., 2013; Dodds et al., 2008; Dufour et al., 2007; Fimm et al., 2006; Manly et al., 2005; Matthias et al., 2010; Newman et al., 2013; Perez et al., 2008, 2009), it would result in the same outcome (disengagement of the ventral network). We therefore conclude that the common likely denominator of rightward shifts with manipulation of both line length and perceptual load/arousal is a disengagement of areas around the right TPJ, in line with the view that downregulation of RH activity leads to a transient change in spatial attentional sampling at the periphery which attenuates the left visual field advantage and causes the observed rightward shifts in bias.

The above interpretation assumes that the line-length effect occurs due to a modulation of brain activity at a higher-order (visuospatial attention) processing stage. Yet, it is important to consider whether the current EEG results may alternatively be explained by a lower-level visual account related to the change in stimulus size (stronger visual evoked response to long relative to short lines). We are confident that this explanation can be discarded given that the observed timing (N1), lateralization (RH only) and localization (TPJ) of the ERP effect is not in line with such a low-level account: If low-level factors alone accounted for the ERP-effect, we would expect differences between long and short lines to onset at an earlier stage of stimulus processing (such as the C1 component), with a more posterior bilateral topography and occipital source estimates (Di Russo et al., 2002; Foxe et al., 2008), and we also would not have expected a correlation with behavioral spatial bias at the latency of the N1 component.

### Early versus late EEG responses in line bisection: stimulus-driven, reflexive vs. task-related, decisional stages of spatial processing

Our results reveal two main ERP-events that are modulated by the experimental manipulations, an early ERP-event occurring at around N1 showing characteristics of an automatic (reflexive) response (occurring independently of task and therefore being primarily stimulus-driven) and a later event depending on task (spatial versus non-spatial line judgments) irrespective of stimulus properties (line length). Importantly, only the first event is correlated with line bisection behavior, while the later is not. This dissociation strongly suggests that the two ERP events reflect different processes in task processing, further corroborated by their right hemispheric lateralization to two distinct sources, areas of the right TPJ versus right superior parietal cortex respectively. Note that the timing and localization of these two ERP-events accord with and extend the findings of Foxe et al. (2003) who report right TPJ source estimates at early phases of line bisection and right superior parietal cortex estimates (in the vicinity of IPS) at later phases, in good agreement with fMRI studies of landmark task processing (Cai et al., 2013; Cavezian et al., 2012; Çiçek et al., 2009; Fink et al., 2000a,b, 2001). It is of interest to note that the time point and topography of the early effect implicate the N1, an early component of the visual ERP implicated in object discrimination and recognition (Allison et al., 1999; Doniger et al., 2001; Vogel and Luck, 2000). In addition, pseudoneglect is stronger for solid continuous lines relative to line endpoint judgment, or lateralized segment distance/size judgments (Post et al., 2001), suggesting that behavioral biases may arise more strongly at an allocentric (object-based) level of processing (Foxe et al., 2003; Singh et al., 2011), or are strongly dependent on stimulus saliency/energy. In line with the latter view, it has been proposed that the strength of engagement of a right lateralized attention system is likely to depend on stimulus properties (see e.g. Benwell et al., 2013; Snyder et al., 2012), such that more salient stimuli (here longer lines) may lead to a stronger engagement of this right hemispheric system, and consequently drive a stronger leftward behavioral bias.

As to the functional role of the task-related, right superior parietal cortex activation, we postulate involvement at a decisional stage of task performance. In line with our finding of late superior parietal cortex/IPS activity-differences, previous imaging studies comparing the landmark task with a non-spatial control task have found modulation of activity to be strongly lateralized to the right superior parietal cortex (in the vicinity of IPS) both using EEG (Foxe et al., 2003), and fMRI (Cai et al., 2013; Cavezian et al., 2012; Çiçek et al., 2009; Fink et al., 2000a,b, 2001). However, these studies were restricted to relatively long line task performance (> 6° horizontal visual angle). In the current study, right hemispheric dominance for landmark task processing was also found for short lines (1° horizontal visual angle) in the absence of any systematic behavioral bias, thus suggesting that the relatively late right hemispheric task effect (peaking at 280 ms post-stimulus onset in the current study and at 310 ms in Foxe et al., 2003) does not represent an activation pattern that can alone explain the genesis of spatial bias. Instead, our finding of a correlation between the strength of RH activation earlier in time (100–200 ms) and the behavioral bias displayed across participants clearly point to an earlier temporal locus of the bias, in line with previous single-pulse TMS studies (Dambeck et al., 2006; Fierro et al., 2001) and ERP studies of visuospatial processing in neglect patients (Di Russo et al., 2008; Tarkka et al., 2011). We therefore speculate that the later, task-related activity may represent more memory rehearsal/decisional stages for task performance that occur after initial attentional engagement (and the accumulation of sensory evidence), and that do not determine the extent of the spatial bias (Duncan, 1980; Luck et al., 2000; Philiastides and Sajda, 2006).

### Future directions

Interestingly, in two recent EEG studies, the rightward shifts in behavioral bias associated with time-on-task (Newman et al., 2013) and increased perceptual load (Perez et al., 2009) have also been linked to changes in oscillatory activity. These studies focused on lateralization of posterior alpha-band activity, which represents a reliable marker of the degree of spatial attentional engagement during anticipatory attention orienting prior to stimulus onset (Foxe and Snyder, 2011; Gould et al., 2011; Kelly et al., 2006; Macdonald et al., 2011; Sauseng et al., 2005; Thut et al., 2006; Worden et al., 2000). An interesting line for future research would be to investigate the relationship between different experimental manipulations of spatial bias and both post-stimulus (as in the current study, O'Connell et al., 2011; Perez et al., 2009) and pre-stimulus EEG activity (Newman et al., 2013), and to establish how these separate EEG measures implicated in the processing of visuospatial information relate to one another in the genesis and modulation of spatial bias.

## Conclusion

The present EEG study has identified the ERP correlate of changes in line bisection bias with manipulation of line length. Our results suggest that the degree to which the right hemispheric ventral attention network is engaged during the early phases of stimulus processing (~100–200 ms post-stimulus onset) modulates the degree of spatial bias displayed across individuals. Further research on experimental manipulations of spatial bias and their EEG correlates may elucidate the role played by attentional subsystems, their interactions and their contribution to the (often biased) distribution of spatial attention in both healthy individuals and post-stroke neglect patients.
